# Cogito, Ergo Contraho: Think Big or Think Small? How Construal Level Theory Shapes Creative Agreements

**DOI:** 10.3390/bs15060775

**Published:** 2025-06-04

**Authors:** Hyeran Choi

**Affiliations:** School of Business Administration, College of Business and Economics, Chung-Ang University, Seoul 06974, Republic of Korea; drchoi@cau.ac.kr; Tel.: +82-02-820-5628

**Keywords:** creative agreements, construal level theory, integrative negotiation, cognitive flexibility, decision-making

## Abstract

Creativity is a vital element for successful negotiation and positive business outcomes. However, a complete understanding of how different thinking styles shape creative agreements is still developing in research. This study examines how broad and detailed thinking affects the ability to create inventive deals, with a particular focus on construal level theory. While past research has often highlighted abstract thinking as the main way to be creative, this study shows that both abstract and concrete thinking contribute to making creative deals but in different ways. For example, Study 1 looks at how concrete thinking helps in forming creative agreements, tested with 114 undergraduate students in a laboratory experiment. This challenges the common idea that concrete thinking limits new ideas. Study 2 then explores the benefits of thinking that combines different viewpoints, where negotiators can skillfully switch between abstract and concrete ways of thinking, tested with 96 students in another laboratory experiment. Across two experiments, mediation analyses were conducted to examine the hypothesized relationships. Findings indicate that cognitive flexibility—the ability to switch thinking styles—benefits both idea generation and specific problem-solving. This study’s implications span negotiation strategies, fostering organizational creativity, and developing flexible thinking approaches for problem-solving.

## 1. Introduction

In business and organizational settings, integrative agreements are essential for fostering long-term collaboration, especially when traditional negotiation strategies fall short of uncovering mutually beneficial outcomes. Integrative agreements reconcile both parties’ interests and lead to higher joint benefit (Follett, 1940; Pruitt, 1983; Walton & McKersie, 1965). There are several ways of achieving integrative agreements, and much of the existing research has focused on the role of tradeoffs across known issues using scorable negotiation tasks. These studies have underscored the importance of sharing information about issue priorities to enable mutually beneficial outcomes (Bazerman et al., 1985; Froman & Cohen, 1970; Pruitt & Lewis, 1975; Thompson, 1991; Thompson & Hastie, 1990). While tradeoffs remain a fundamental pathway to integrative agreements, recent research has increasingly focused on creativity in negotiation—particularly the process of discovering or introducing new issues (Curhan et al., 2021; Fousiani et al., 2022; Yao et al., 2020). This study follows a new idea and investigates other thinking methods that can help negotiators develop creative solutions, especially when issues are unclear or new solutions are possible. To do so, parties might identify and add new issues (e.g., Fousiani et al., 2022; Kray et al., 2009) or they might unbundle an existing issue (e.g., Butler, 1994; Jang et al., 2021). To understand the cognitive mechanisms driving negotiation creativity, this study draws on psychological principles while analyzing this vital business endeavor. Rather than focusing on conventional success metrics like quantifiable outcomes, this work concentrates on identifying the cognitive paths that lead negotiators to formulate and recognize uniquely innovative solutions.

The conditions examined in this study represent distinct approaches to processing negotiation situations, rooted in construal level theory (CLT) (Liberman & Trope, 1998; Trope & Liberman, 2010). CLT suggests that the way people think about and understand things—whether as abstract ideas or concrete details—forms a kind of scale. When thinking abstractly, also known as high-level construal, individuals tend to focus on the main purposes and important values. On the other hand, concrete thinking, or low-level construal, puts more emphasis on the specific parts and the actions that can be taken. For example, our sense of when something will happen can influence this; things far in the future often are thought about in a more general way, while things happening soon are considered with more specific details in mind.

Across different areas of management, including leadership, organizational change, employee involvement, integrative agreements, and consumer behavior, the usefulness of CLT has been looked at more and more (e.g., Berson et al., 2021; Chen et al., 2023; Giacomantonio et al., 2010a, 2010b; Goodman & Malkoc, 2012; Luan et al., 2023; Park et al., 2025; Wening et al., 2015). These studies suggest that CLT may offer valuable insights into strategic thinking and the functioning of organizations.

For negotiation, this study suggests that, when negotiators focus their thinking on the small details (low-level construal), it could help them to understand the specific issues better (c.f., Giacomantonio et al., 2010a). This better understanding might then allow them to break down and change the issues. Also, being able to switch without difficulty between thinking about the big picture and the small details—we often call this cognitive flexibility—would help in obtaining more creative and better results for both sides. To examine these ideas, two experiments were conducted in which participants were instructed to think either in a general or a specific way or to alternate between the two, and the impact of these thought processes on negotiation outcomes was observed.

## 2. Theoretical Background and Hypothesis

### 2.1. Creative Agreements

Creative agreements in negotiation are those outcomes distinguished by a redefinition of the core issues under discussion (Putnam & Holmer, 1992). The negotiation literature has identified two main types of creative agreements: “unbundling” and “adding” (Lax & Sebenius, 1986, 2006; Pruitt, 1981; Thompson, 1998). Unbundling, which is also called fractionation (Fisher, 1964) or unlinking (Pruitt, 1981; Pruitt & Carnevale, 1993), involves separating an issue into components, and then certain components are traded, altered, or dropped. A classic example of unbundling is Follett’s (1940) anecdote about two sisters and an orange. Instead of cutting it in half, they broke the fruit into its different components, with one sister using the peel for baking and the other using the juice for drinking. Negotiations between Egypt and Israel over the Sinai are a real-world example of unbundling (Fisher et al., 2011). Their negotiations initially presented as a zero-sum scenario if the parties were to contend over the simple percentage of land each would obtain. In such a case, every square mile gained by one party would directly translate to a square mile lost by the other. However, a significant departure from this model occurred through the unbundling of the Sinai: Egypt sought sovereignty and was granted the land’s return, while Israel desired security, leading to the land’s demilitarization. As seen in both the orange example and the Sinai example, the general approach to creating value by unbundling is to break down an original issue into components so that the components can be traded off or discarded, leaving parties with more of what they want and less of what they do not want.

There are few empirical studies examining unbundling. A study by Jang et al. (2021) demonstrated that, when situational constraints made reaching a simple price agreement infeasible, negotiators were more likely to pursue integrative agreements—including customized modifications of the original issue—as a way to overcome impasses. Another study by Butler (1999) showed that increasing trust led to increased information exchange and increased rates of creative agreements due to unbundling. An unpublished study by J. Loewenstein and Howell (2010) showed that an increased understanding of one’s own interests led to increased rates of unbundling an existing issue (J. Loewenstein & Howell, 2010).

Adding issues means negotiators find and bring in new topics that were not talked about at the start. For example, while talking about how much a product costs, a buyer might suggest adding a guarantee. This could happen after the buyer sees the seller is sure about the product’s quality and the buyer is worried about the price because they are not sure if the product will last.

Adding new issues is commonly seen in negotiations. For example, job negotiations frequently consider new terms such as work–life balance, spousal hires, educational leave, clothing allowances, travel support, and so forth (Rousseau, 2001; Wronowska, 2023). Several empirical papers have used negotiation situations that allow for adding new issues to the discussion. For instance, when negotiators take the other party’s perspective, they appear to be more likely to add new issues (Galinsky et al., 2008). Also, negotiators need both the ability to add new issues and the cognitive flexibility to integrate them creatively while mental fatigue hinders this process (Zhang et al., 2025). In addition, in a cooperative context, criticism facilitated the generation of more new issues among negotiators (Curhan et al., 2021). Another paper found that strategic behavioral mimicry helps to build trust, increase information sharing, and thereby increase the rate of adding new issues to create value (Maddux et al., 2008). Positive affect also appears to increase trust and information sharing and, as a result, leads to adding a new issue to create value (Anderson & Thompson, 2004). Thus, there is some evidence that trust and information sharing are relevant to both unbundling issues and adding issues to create value. However, none of this research has focused on what specifically is involved in unbundling issues versus adding issues.

Previous work may assume that the same factors lead to forming any integrative agreement. Negotiators are typically encouraged to share high-level information about their interests, such as their overarching preferences or priorities, rather than focusing on the details of each issue as the means to generate integrative agreements (e.g., Benetti et al., 2021; Thompson, 1991; Weingart et al., 2007). Sharing broad, high-level information presumably would be helpful for understanding the big picture of the negotiation situation. Parties should be stepping back from the immediately available issues and thinking about the broader situation. As new issues external to the already apparent ones are presumably only to be found by thinking beyond what is already under discussion, thinking about the broader situation could be useful for promoting the addition of new issues. However, it is not clear if that would be helpful for unbundling issues. In addition, individuals with a high construal level may vary in the degree of abstraction. Thus, focusing on issues of value creation between parties could present challenges, as abstract concepts tend to be more indefinite, while concrete ones are more definite (cf. Flesch, 1950). To address this, the present work begins by emphasizing a lower-level construal of value creation, where concrete, issue-level thinking can help clarify and operationalize discussable components of the negotiation.

Unbundling issues would seem to require understanding what specific, detailed elements of the issues are necessary or unnecessary to customize the best terms for the parties. Stepping back from the available issues and thinking of the big picture would presumably not foster unbundling them but turning to alternative new issues instead. The first step to unbundling is presumably examining components of the existing issue and analyzing each component to identify its role and value. Following these steps, parties may be able to find which components are helpful to meet their interests and which components are not necessary for their interests. By analyzing the components, parties could disaggregate an existing issue, redefine it, and so form creative agreements.

### 2.2. Low Construal Level for Creative Agreements

As noted earlier, CLT suggests that individuals construe psychologically close or immediate events more concretely, while distant events are construed more abstractly (Liberman & Trope, 1998). The present research focuses on how a concrete thinking style (low-level construal) affects negotiation outcomes. Previous studies, like the one by Shani et al. (2009), show that people who think in a general way (high-level construal) do not tend to look closely at the specific details of information as much as people who think in a concrete way. Similarly, Alter et al. (2010) found that people who think in a general way often focus on the main use or purpose of something and often neglect specific components. Also, Förster et al. (2004) reported that, when people were encouraged to think in a concrete way, they did better on tasks that needed them to analyze specific, detailed information compared to when they were encouraged to think in a general way. This kind of task needs very close attention to small details and, for such tasks, a low-level construal is more effective than a high-level one (Pfeiffer et al., 2014). In negotiation, carefully looking at all the important information using low-level construal can be a helpful step to understanding the existing negotiation issues. On the other hand, relying on high-level construal might not be as helpful for this kind of detailed examination. In short, negotiators who tend to use low-level construal will adopt a more detail-oriented focus and approach, as they are more attuned to specific information.

Often, the way a negotiation will end up is largely decided in the time leading up to the formal exchange of proposals, with the planning phase acting as a very important foundation for the entire process. Effective planning allows negotiators to analyze their own interests, understand the structure of the issues at stake, and anticipate the counterpart’s priorities (Thompson, 1991; Lax & Sebenius, 1986). Negotiation scholars consistently emphasize that negotiators who engage in thorough planning are better equipped to identify key issues (Gelfand & Brett, 2004; Thompson, 1991). Aligned with this, empirical research shows that thorough preparation significantly enhances negotiation outcomes (Dudek & Stadtler, 2005; Simosi et al., 2021). Given that preparing for a negotiation demands considerable mental effort and greatly influences subsequent discussions, the way negotiators frame the situation during the planning phase can shape the types of agreements they ultimately reach.

Taken together, since planning is the starting point of any negotiation, the construal level adopted during this phase can significantly shape how negotiators approach the process. Negotiators with a low-level construal are more likely to focus on concrete details and specific features of the issues at hand, rather than abstract, overarching goals. This detailed focus is likely to lead to more specific planning notes, emphasizing actionable and discussable components. Thus, the following hypothesis is proposed:

**Hypothesis** **1a.** *Negotiators with low-level construals will exhibit a greater focus on concrete, issue-specific details during planning than those with high-level construals.*

Focusing on the details of existing issues during negotiation planning can have significant consequences. The ability to unbundle issues depends on negotiators identifying key information within components, such as latent attributes or divisible sub-elements not easily seen from a holistic view. This information resides in the concrete specifics that allow an issue to be disaggregated or reframed for value creation. For instance, understanding cost structures or timelines can reveal trade-offs or tailored agreements that might otherwise be overlooked. Consequently, negotiators who focus on these concrete details are more likely to identify unbundling opportunities. Thus, greater attention to issue-specific details increases the likelihood of recognizing key factors that enable issue unbundling during planning. Based on this, the following hypothesis is proposed:

**Hypothesis** **1b.** *A focus on concrete issue details will be positively associated with identifying key information to unbundle existing issues.*

Having considered the key information for unbundling during planning should, in turn, make parties more likely to make use of that information during their negotiations. Specifically, parties should be more likely to form an agreement that unbundles an existing issue if they have noted the potential relevance of a detail of the main issue during planning. More generally, negotiators who focus on the key information for unbundling during their planning could be expected to be particularly likely to craft a deal that involves issue restructuring. Thus, the following hypothesis is proposed:

**Hypothesis** **1c.** *Identifying key information relevant to unbundling during the planning phase will be more likely to form integrative agreements that involve issue unbundling.*

**Figure 1:**
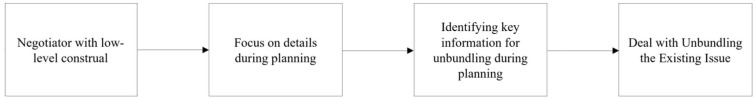
Conceptual model linking construal level to creative agreements through planning focus and issue unbundling.

These theoretical relationships are summarized in the conceptual model (Figure 1), which visually outlines the proposed pathways.

### 2.3. Mixed Construal Levels and Creative Agreements

If low-level construals facilitate the unbundling of existing issues—whereas high-level construals alone may be less effective due to abstract-level mismatches between parties—a natural next question is whether negotiators can benefit from flexibly shifting between construal levels to form creative agreements that both unbundle and add issues. From a prescriptive standpoint, this ability would be highly advantageous, allowing negotiators to maximize value creation by leveraging both abstract and concrete perspectives.

To achieve this balance, the pair of negotiators needs to work together to use both big-picture thinking and detailed thinking. This helps them fully understand both what is really important to each side and the specific issues being discussed. This can happen mainly in two ways. First, each negotiator can focus on a different level of thinking—one thinks about the overall goals, while the other focuses on the specific details. This way, they bring different but helpful viewpoints to the negotiation.

The second way, and the one suggested in this study, is when each negotiator can think in both big-picture and detailed ways on their own. They can also switch between these ways of thinking as they plan and negotiate. This ability for each negotiator to see things from different levels helps everyone understand the situation better, reduces the chance of misunderstandings, and makes it more likely they will reach agreements that work well for both sides.

This proposition builds on prior research suggesting that, even when important information is shared, it may go unused if the negotiation context is ambiguous or complex (G. Loewenstein & Moore, 2004). When the people negotiating are thinking on different levels—one focused on the big picture and the other on the details (the first situation)—information the other side gives might seem unimportant or unclear. This could lead to them not using that information well during negotiation. However, when each person can flexibly switch between thinking about the big picture and the specific details (the second situation), they are better able to understand and use both kinds of information. This better way of processing information can help them come up with more creative agreements. Other research has also found evidence for this idea.

In experiential learning theory (Kolb, 1984), learning and adaptation are optimized through iterative movement between concrete experience and abstract conceptualization. This back-and-forth between different ways of thinking improves understanding and innovation by encouraging both being aware of the specific situation and thinking about it in a new, more general way—both of which are important in creative negotiation. In teaching, instructors often switch between general rules and specific examples to help students understand and use what they learn in new situations. Research on wise reasoning (Grossmann et al., 2016) further supports this view: individuals who demonstrate cognitive flexibility are more likely to engage in open-minded, reflective, and socially attuned reasoning.

Consistent with these findings, a construal level shift model (Park et al., 2025; Steinbach et al., 2019; Wiesenfeld et al., 2017) argues that both concrete and abstract thinking are needed for comprehensive problem formulation but at different stages. According to this theory, the level of abstraction in our thinking—whether abstract or concrete—influences both the aspects of information we attend to and the way we engage in reasoning. For instance, when implementing a plan, it is often necessary to concentrate on specific, detailed elements. However, periodically shifting to a more abstract, high-level perspective is also essential in order to maintain alignment with overarching strategic objectives. This dynamic perspective underscores the value of sequencing and flexibility rather than rigidly favoring one mode of thinking over the other (Park et al., 2025).

When each negotiator can switch between broad and detailed thinking during planning, it will help them understand the negotiation situation in a more balanced and complete way. It would help them to put together both the main goals and the specific details. This, in turn, helps guide how they talk and makes it more likely they will find chances to add new issues and break down existing ones.

On the other hand, dyads in which each negotiator has different thinking styles—one uses broad thinking and the other uses detailed thinking—may face challenges in coordination due to divergent areas of focus. Each negotiator might pay attention to different kinds of information, with one stressing the main goals and what they want in the long run, while the other focuses on the small details of the issues. This mismatch can cause misunderstandings, result in fragmented dialogue, or lead to missed opportunities, especially if they do not see why the other party’s ideas are important. Thus, they might find it hard to combine their viewpoints during the negotiation, which can limit how well they can come up with creative agreements. Synthesizing these ideas, the following hypothesis is proposed:

**Hypothesis** **2a.** *Dyads in which each negotiator flexibly engages with both high-level and low-level construals (i.e., within-party construal flexibility) will be more likely to generate creative agreements that involve both the addition of new issues and the unbundling of existing ones, compared to dyads in which each negotiator maintains different fixed construal levels (i.e., between-party construal flexibility).*

Since Hypothesis 2a posits that different combinations of broad and detailed thinking between negotiators affect the results of the negotiation, this study also examines the cognitive and interactional processes that may account for these effects. The main idea in Hypothesis 2a is that negotiators who use both broad and detailed thinking will plan differently from those who only use one type of thinking. As previously noted, adhering to a single mode of thinking will probably make negotiators focus either on the general, goal-related situation (broad thinking; high-level construal) or on the specific details of the issues (detailed thinking; low-level construal).

The concurrent application of abstract and concrete cognitive modes, conversely, empowers negotiators to engage with diverse information. Abstract thought supports concentration on strategic aims, whereas concrete thought permits a thorough investigation of issue specifics. This fluid transition between cognitive levels cultivates cognitive flexibility, enabling simultaneous management of core interests and the discrete elements of the ongoing discussion. This approach aligns with research on cognitive flexibility, which emphasizes the value of shifting between different mental representations to solve complex problems (Maddux & Galinsky, 2009; Maddux et al., 2010). It also echoes the logic of experiential learning theory, which highlights the importance of moving between abstract conceptualization and concrete experience for effective decision-making (Kolb, 1984), as well as the construal level shift model (Park et al., 2025; Steinbach et al., 2019). Thus, the following hypothesis is proposed:

**Hypothesis** **2b.** *Dyads in which each negotiator flexibly engages with both high-level and low-level construals (i.e., within-party construal flexibility) will be more likely to incorporate both underlying interests and detailed issue-specific information during planning, compared to dyads in which each negotiator adopts different fixed construal levels (i.e., between-party construal flexibility).*

The information on which parties focus during planning is likely to shape the discussions they have during negotiation. Planning helps negotiators organize their thoughts, anticipate counterpart responses, and prioritize what information to surface (Gelfand & Brett, 2004; Thompson, 1991). Therefore, when negotiators focus on the broad background situation—such as long-term goals, interests, or values—they are more likely to bring this abstract perspective into the conversation and introduce new issues that align with those broader aims. Conversely, when negotiators concentrate on the detailed features of the existing issues during planning, they are more likely to identify specific components that can be restructured and to raise these details in discussion, facilitating issue unbundling.

Dyads in which parties adopt both high-level and low-level construals during planning—thus recognizing information about both overarching interests and specific issue elements—are particularly likely to engage in negotiation discussions that incorporate both types of key information: that which supports the addition of new issues and that which facilitates restructuring the existing ones. Thus, the following hypothesis is proposed:

**Hypothesis** **2c.** *Incorporating both underlying interests and detailed issue-specific information during planning will be positively associated with sharing key information to add and unbundle issues during discussions.*

Negotiation discussions are predictive of the types of agreements that parties ultimately form, as shown in longstanding research on information sharing and integrative outcomes (e.g., Fisher et al., 2011; Pruitt, 1981). For example, Weingart et al. (2007) demonstrated that different types of information exchange produce different negotiation results. Their process analysis revealed that sharing information about preferences and priorities increased the likelihood of integrative agreements (e.g., trade-offs), whereas exchanging factual information tended to lead to distributive outcomes.

Following this logic, if parties discuss key information related to the broad aims of the negotiation—such as long-term interests or higher-order goals—they are more likely to generate agreements that incorporate newly added issues satisfying those interests. Similarly, if parties discuss key details of the existing issue structure, they are more likely to recognize opportunities for unbundling and restructuring them. Accordingly, parties who discuss both types of key information—abstract aims and concrete issue features—are especially likely to form creative, integrative agreements that reflect both the addition of new issues and the unbundling of existing ones. Thus, the following hypothesis is proposed:

**Hypothesis** **2d.** *Negotiation discussions that include key information to add and unbundle issues will be positively associated with the formation of creative agreements that reflect both types of value creation.*

**Figure 2:**
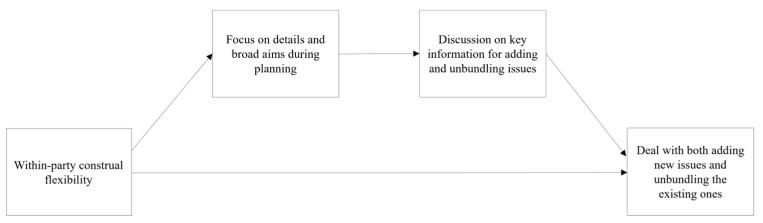
Conceptual model of within-party construal flexibility and creative agreement formation.

These theoretical relationships are summarized in the conceptual model (Figure 2), which visually illustrates the proposed pathways.

## 3. Experiments: Methodology, Materials, and Results

Overview of the Current Experiments

This study used controlled laboratory experiments to examine how abstract and concrete thinking influence the development of creative agreements. The first experiment investigated how a detail-focused cognitive style (low-level construal) facilitates innovative negotiation outcomes. The second explored how negotiators who flexibly shift between broad and detailed thinking create value by both introducing new issues and unbundling existing ones.

The laboratory setting was critical, allowing precise manipulation of construal levels and close observation of negotiation dynamics. It enabled tasks designed to elicit specific cognitive styles, detailed recording of negotiation dialogues, and tight control over contextual variables. A pilot simulation ensured that the two value-creation pathways—issue addition and unbundling—were equally challenging and that the task context was suitable for participants.

The author used ChatGPT (OpenAI, GPT-4) to support language refinement and clarity.

### 3.1. Experiment 1: Construal Level as an Influence on Unbundling

Experiment 1 tested three initial hypotheses, centered on the idea that individuals induced into a concrete, detail-oriented mindset (low-level construal) would more thoroughly examine the specific attributes of an existing issue during preparation. This heightened attention to detail was expected to enhance their ability to identify key restructuring opportunities, thereby increasing the likelihood of generating value through unbundling.

#### 3.1.1. Method

Participants and design. A total of 114 undergraduate students from a large Midwestern university in the United States participated in the study. Participants were recruited through the business school’s subject pool and received extra credit for their participation in a course. In the study, participants were randomly paired into dyads. Each dyad was randomly assigned to one of two construal conditions: low-level construal (*N* = 58) or high-level construal (*N* = 56).

While the sample consisted of undergraduate students, which may limit the generalizability of the findings to other populations, this sample is considered appropriate for the laboratory-based nature of the study (c.f., De Dreu & Carnevale, 2005). Additionally, the random assignment to conditions helps mitigate potential biases in the experimental process. The sample size was determined based on previous research using similar experimental designs (e.g., Bar-Anan et al., 2006; Galinsky et al., 2005), and power analyses indicated that this sample size was adequate to detect significant effects.

Negotiation task. This study used a two-person negotiation exercise involving a tenant and a potential subtenant discussing a summer sublease for an apartment. The scenario was chosen because it is a familiar situation that participants could easily relate to. The author of this paper contributed to designing the simulation, ensuring it provided opportunities for value creation—by introducing new issues and breaking down existing ones into smaller, manageable parts.

In this scenario, the tenant is a graduating student leaving for the summer and looking to sublet their studio apartment. They have advertised the unit and are aiming to minimize financial loss while also managing end-of-term tasks. The prospective subtenant, needing short-term housing near campus, has their own budget and preferences. The tenant’s asking price, however, is higher than what the subtenant can afford, making a simple price agreement impractical for both.

The goal of the negotiation was not just to bargain over price. It allowed the parties to break the rental agreement into different components, such as selling the parking space separately or adding new issues like the tenant selling textbooks to the subtenant. These opportunities for creating value were built into the materials provided to participants. In the scenario, the different agreements are readily quantified: price–deal, impasse, unbundling, adding, and having both unbundling and adding.

Procedure. Participants signed up for the study online. Upon their arrival at the laboratory, they were randomly assigned to dyads, conditions, and roles. Each dyad worked in its own room. Participants were seated separately in front of computers. All instructions were presented on computers, with the exception of negotiation role materials, which they received on paper. Participants began by completing the construal level manipulation tasks, followed by reading the materials outlining their specific role in the negotiation scenario, and then proceeded with their individual negotiation planning. When both parties were ready to negotiate, they moved to a table away from the computers and the experimenter turned on the recording equipment and then left the room while they held their discussions. After negotiating, participants returned to their computers to report their negotiation agreements and completed the Subjective Value Inventory (SVI; Curhan et al., 2006), which measures negotiation satisfaction, interpersonal rapport, and self-impressions during the negotiation, along with demographic questions. Then, they were debriefed, thanked, and allowed to leave.

Data analysis plan. The data were analyzed using SPSS (Version 24.0) to obtain basic statistics and to test the hypotheses. Hayes’s (2017) PROCESS macro (Model 6) was used to test the suggested mediation model. Specifically, independent-samples t-tests were used to check if the manipulation worked and to compare the differences between the groups (low-level construal versus high-level construal) in terms of the length of participants’ descriptions on the construal manipulation task, how much they focused on details while negotiation planning, and how much value they created under different thinking conditions. Mediation analysis was conducted to test if the effect of broad or detailed thinking on breaking down agreements happened indirectly through the details of the issue and the key details for unbundling. The assumptions for the t-tests were checked, and multicollinearity was assessed for the mediation analysis.

Construal-level manipulation. Adapting materials developed by Alter et al. (2010), construal level was manipulated by asking participants to complete a mindset prime. The low-construal-level condition was coded as “0” and the high-construal-level condition was coded as “1”.

Manipulation check. To assess the effectiveness of the construal-level manipulation, the number of words participants wrote during the planning phase to describe their negotiation was counted. According to CLT, individuals with a low-level construal tend to focus on concrete details and therefore may use more words, while those with a high-level construal focus on broader abstractions and may use fewer words. Thus, the length of participants’ descriptions served as a manipulation check for construal level.

Focus on details. The number of descriptions regarding the details of the existing issue (e.g., price, rental duration, and utility fees) was counted in participants’ descriptions of the case during their planning to measure their focus on the existing issue.

Unbundling Focus Index. The index was generated to capture the number of statements regarding key information for unbundling the existing issue (e.g., recognizing the parking space or no car) counted during planning to assess participants’ focus on unbundling opportunities.

Unbundling. If the terms of the agreement include unbundling (e.g., selling the parking space to a third party), the outcome was coded as “1” and “0” if unbundling was not included.

Value created. Using the case materials and the terms of the agreements, the total value created by an impasse was scored as USD 0, a simple price deal as -USD 100, a deal with unbundling as USD 80, a deal with adding as USD 80, and a deal with both adding and unbundling as USD 260.

Negotiation process measures. Two coders, who were trained on a separate negotiation scenario prior to the study, listened to each negotiation and recorded every offer, exchange of information related to the items linked to the details, key items for unbundling the existing issue, items linked to the broad aims (e.g., “no need to live here” or “need somewhere to live for the summer semester”), and key items for adding new issues (e.g., buying or selling textbooks). Inter-rater reliabilities were all above 0.75.

#### 3.1.2. Results

Manipulation check. Those in the low-construal-level condition (M = 293.17, SD = 148.21) tended to write longer descriptions of the case during planning than those in the high-construal-level condition (M = 219.68, SD = 123.43), *t* (112) = 2.87, *p* < 0.01. This is evidence that the construal manipulations successfully induced the different construal levels.

The low construal to the unbundling pathway. Supporting Hypothesis 1a, construal level was associated with writing about the details of the existing issue (*r* = −0.42, *p* < 0.05), as those in the low-construal-level condition tended to provide more statements about items linked to the apartment sublease (M = 2.21, SD = 1.82) than dyads in the high-construal-level condition (M = 0.89, SD = 0.96), *t* (55) = 3.39, *p* < 0.05.

**Figure 3:**
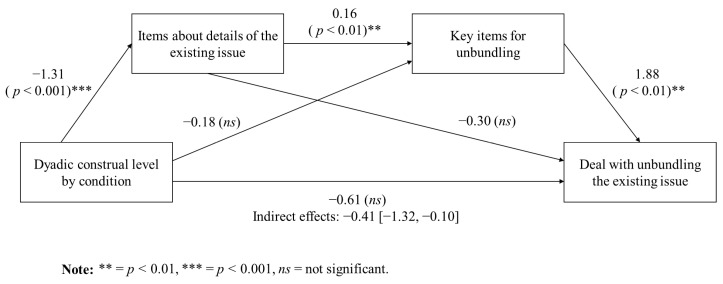
Indirect effects of construal level on a deal with unbundling through planning about the existing issue in Experiment 1.

Writing more statements about details of the existing issue was, in turn, associated with writing about the key items for unbundling (*r* = 0.46, *p* < 0.05). Focusing on the key items for unbundling during planning was then correlated with forming deals that unbundled the main issue (*r* = 0.46, *p* < 0.05). As this pattern indicates, and as confirmed by a mediation analysis using Hayes’s (2017) PROCESS macro (Model 6), there is a reliable indirect effect of construal level, through details and key details, on forming an agreement with unbundling, B = −0.42 (95% bootstrap confidence interval from −1.32 to −0.10). Figure 3 shows the full path model showing that low construal levels lead to more statements about details of the existing issue during planning, which, in turn, leads to more statements about key details for unbundling the existing issue during planning and ultimately culminating in the generation of a deal that unbundles the existing issue. Thus, Hypotheses 1a, 1b, and 1c were supported.

The mediation analysis revealed a statistically significant indirect effect of construal level on the extent to which issues were unbundled. This effect was mediated by the level of detail that participants considered regarding the existing negotiation issues (indirect effect = −0.41, 95% CI [−1.32, −0.10]). Specifically, those in the low-construal-level condition exhibited a stronger tendency to focus on the particular components of the negotiation (such as utility fees or items included with the apartment) when compared to participants in the high-construal-level condition (*β* = −1.31, *p* < 0.001). This increased attention to specific details subsequently enhanced the probability of participants identifying key information relevant to unbundling issues (*β* = 0.16, *p* < 0.01), which, in turn, led to a greater likelihood of reaching a final agreement that involved the unbundling of issues (*β* = 1.88, *p* < 0.01). Results suggest that a negotiator’s construal level shapes strategic orientation. By guiding cognitive focus towards specific aspects of the issues, it enables the formation of value-adding agreements, such as through unbundling.

#### 3.1.3. Additional Analysis

Value created. As expected, parties who formed agreements that included unbundling the main issue tended to create more value (M = USD 117.50, SD = USD 74.67) than parties who did not (M = −USD 35.76, SD = USD 80.12), *t* (55) = 7.34, *p* < 0.001.

Negotiation process. An examination of the transcripts showed several trends. In both conditions, the early phase of the negotiations sounded alike. Negotiators mostly just negotiated prices, and the initial prices were comparable in the two conditions. First offers were nearly exclusively price offers (98%; 56 out of 57) rather than including value-creating options. Thus, although parties noted key information during planning, this did not result in opening discussions of such information.

Parties did eventually discuss key information. There was no difference due to construal condition of sharing the key information for unbundling or adding. Surprisingly, dyads in the low-construal-level condition shared more information about the broad aims than the dyads in the high-construal-level condition, (M_LowCL_ = 2.21, SD_LowCL_ = 1.84; M_HighCL_ = 1.36 SD_HighCL_ = 1.13), *t* (55) = 2.09, *p* < 0.05. A further observation is that the vast majority of the dyads shared information about the key information for unbundling (27 out of 29 dyads in the low-construal-level condition; 27 out of 28 dyads in the high-construal-level condition) and the key information for adding (23 out of 29 dyads in the low-construal-level condition; 22 out of 28 dyads in the high-construal-level condition). Thus, sharing information was not the key to generating creative agreements. This aligns with previous research that it is not merely information sharing that matters (G. Loewenstein & Moore, 2004) but rather how parties focus on or attend to that information during the negotiation process. To sum up, the results of Experiment 1 showed that detailed thinking can help in making more creative agreements by making people focus on the small points, like breaking down the original issue.

### 3.2. Experiment 2: Within-Party Construal Flexibility

If different construal levels might foster different outcomes, then it raises the question of how parties might form deals that include more than one type of outcome. In Experiment 1, just 9% of the dyads both added and unbundled issues. Yet it is possible for people to consider the same situation using both a high-level construal and a low-level construal, one after another. The question is whether doing so might enable people to form agreements that both add and unbundle issues.

As negotiations involve more than one person, there are two ways to examine the application of multiple construal levels. One party could adopt one construal and the other party could adopt the other. Or each party could consider the negotiation situation from both construal levels. This within-party construal flexibility (the second scenario) might encourage each person to consider a full range of information, which is likely to be beneficial. While different construal levels across parties (the first scenario) might simply lead to misunderstandings or not finding relevant what the other party is discussing. Experiment 2 examines these two conditions, within-party construal flexibility vs. between-party construal flexibility, and whether each leads to forming deals that both add and unbundle issues.

#### 3.2.1. Method

Participants and design. A total of 96 undergraduate students from a large Midwestern university in the United States participated in the study. Participants were randomly assigned to a dyad and a role. Each dyad was randomly assigned to either the within-party construal flexibility condition (*N* = 48) or the between-party construal flexibility condition (*N* = 48).

Procedure. The procedure in Experiment 2 was identical to that of Experiment 1.

Data analysis plan. The data preparation process began with an initial screening using SPSS (Version 24.0) to identify outliers, handle missing values, and address any data inconsistencies.

Descriptive statistics were first reviewed in Mplus (Version 8.0), which was also used as the main platform for conducting the mediation analysis. Before performing independent-samples *t*-tests in SPSS, the assumptions of normality and homogeneity of variance were examined to ensure appropriateness of the analysis. Multicollinearity was also checked to confirm that the variables were sufficiently independent for the mediation model. Once these preliminary checks were completed, independent-samples *t*-tests were conducted to compare the value created between negotiating pairs. Lastly, mediation analysis was carried out in Mplus to explore whether planning statements and the sharing of key information helped explain the hypothesized mechanisms.

Construal level manipulation. To examine how varying combinations of construal levels influence negotiation processes, two experimental conditions were established: one where each negotiator was encouraged to alternate their thinking modes (within-party construal flexibility) and another where the two negotiators in a dyad were assigned different thinking modes (between-party construal flexibility).

In the within-party construal flexibility group, participants engaged with a modified set of tasks from Experiment 1 (adapted from Alter et al., 2010). To ensure a consistent cognitive load and overall task burden across conditions, participants in the within-party flexibility group completed fewer construal induction exercises—both high and low level—performing half the number of tasks used in the between-party condition. This ensured an equivalent total number of manipulation questions. These participants were also instructed to divide their planning time equally between abstract and concrete cognitive processing, aligning cognitive effort across conditions.

In contrast, in the between-party flexibility group, one participant completed only the high-level construal exercise, while their partner completed only the low-level version, using the same materials as in Experiment 1. This design carefully controlled for total workload and planning time. The goal was to isolate the effect of within-individual variation in thinking style, minimizing potential confounds such as preparation time or task complexity.

Planning Consideration Index. This study focuses on the outcome of both adding and unbundling issues. The primary question was whether each party considered information about the broad goals of the case and the specific details of the main issue during planning. The Planning Consideration Index was generated to reflect the extent to which dyads considered both types of information—related to adding new issues and restructuring existing ones—during their planning.

Each dyad was coded from 0 to 4 as follows: 0 if neither party considered either type of information (broad aims or detailed issue information); 1 if only one party considered one type of information or both parties considered the same single type; 2 if one party considered both types or each party considered a different single type; 3 if one party considered both types while the other considered only one; and 4 if both parties considered both types of information.

Negotiation Key Information Sharing. This reflects the number of key pieces of information regarding adding and unbundling issues that were shared during the negotiation. The information was counted from the negotiation transcripts until one party made an offer that proposed a creative agreement (e.g., proposing selling or buying the textbooks, selling the parking space to the third party, or both).

Deal with both adding and unbundling. If the terms of the agreement include both adding and unbundling options, the outcome was coded as “1” and “0” if both options were not included.

Value created. The outcomes were scored as in Experiment 1.

#### 3.2.2. Results

Deal with both adding and unbundling. Dyads of within-party construal flexibility (25%; 6 out of 24 dyads) generated deals that both added and unbundled issues more than dyads of between-party construal flexibility (0%; 0 out of 24 dyads), χ^2^ (1, *N* = 48) = 6.86, *p* < 0.01, ϕ = 0.38, supporting Hypothesis 2a.

Dyads of within-party construal flexibility to a deal with both adding and unbundling pathway. Dyads assigned to the within-party construal flexibility condition were more likely to reach agreements that involved both the addition of new issues and the unbundling of existing ones. Mixed construal levels within each party, compared to between parties, showed a marginal trend toward greater integration of both abstract and concrete information during the planning stage (*r* = 0.27, *p* < 0.10). Specifically, 75% of dyads in the within-party flexibility condition included both types of information—broad negotiation aims and detailed issue-specific information—whereas only 46% of dyads in the between-party flexibility condition did so.

**Figure 4:**
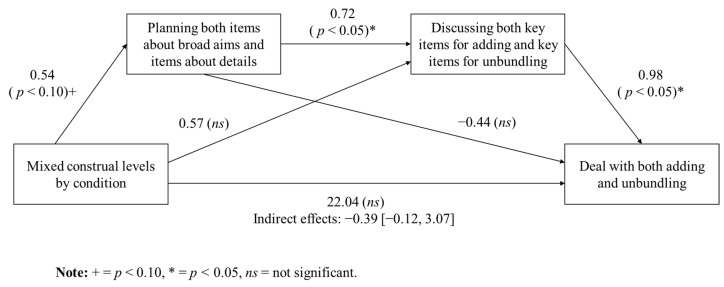
Indirect effects of within-party construal flexibility on a deal with both adding and unbundling through planning and discussion in Experiment 2.

Incorporating both abstract and concrete information during planning was significantly correlated with the sharing of key information during negotiation related to both the addition of new issues and the unbundling of existing ones (*r* = 0.36, *p* < 0.05). In turn, sharing both types of key information during negotiation was positively associated with forming agreements that included both creative pathways (*r* = 0.47, *p* < 0.01). To test the indirect pathway linking construal flexibility to creative agreement-making, a mediation analysis was conducted using Mplus. Although the analysis revealed no statistically significant indirect effect from within-party construal flexibility through planning and information sharing to joint creative outcomes, B = 0.39 (95% bootstrap confidence interval from −0.12 to 3.07), the pattern of results was directionally consistent with the proposed model. Figure 4 presents the full path model, illustrating that dyads in the within-party construal flexibility condition showed a marginal trend toward more integrative planning, which, in turn, led to greater sharing of key information and ultimately to the generation of agreements involving both issue addition and unbundling. Taken together, these results provide empirical support for Hypotheses 2b, 2c, and 2d.

Value created. Dyads with within-party construal flexibility (M_MCL within_ = USD 76.67, SD = USD 26.75) formed more valuable agreements than dyads with between-party construal flexibility (M_MCL between_ = USD 1.67, SD = USD 18.21), *t* (46) = 2.32, *p* < 0.05. This outcome stemmed from more agreements with both adding and unbundling and fewer agreements that were just focused on price.

### 3.3. Summary and Interpretation of Results

Experiment 1 found that low construal levels could influence the formation of creative agreements by unbundling the existing issue. Most prior research has examined factors that influence forming integrative agreements without respect to the kind of integrative agreement (c.f., Barry & Friedman, 1998; Curhan et al., 2021; Jang et al., 2021; Fousiani et al., 2022; Gelfand et al., 2015; Sinaceur et al., 2013). Consequently, this finding is notable in that it provides evidence that a specific factor (in this case, low-level construal) can lead to forming a specific kind of integrative agreement (i.e., unbundling). 

Experiment 2 found that flexibly engaging in both high and low construal levels fostered both adding and unbundling issues. In contrast, a negotiation between parties who each adopted a different construal level did not appear to confer benefits (or generate confusion). There were signs that the reason for the difference was that adopting both high and low construal levels individually encouraged parties to think about both broad aims and detailed information during planning and to discuss both kinds of information in their negotiations.

Although flexibly shifting between high- and low-level construals can require considerable cognitive effort, that this difficulty had a benefit is consistent with previous research that processing information with more effort leads to deeper and more elaborate evaluations of information and better decision-making (De Dreu et al., 2008; Fisk et al., 1999). The cognitive challenge involved in adopting both construal levels aligns with the construal shift model, which emphasizes the importance of moving between abstract and concrete perspectives for strategic achievement (Park et al., 2025; Steinbach et al., 2019) and with Kolb’s experiential learning theory (Kolb, 1984), which highlights the value of alternating between concrete experience and abstract conceptualization to deepen understanding. Importantly, the kind of difficulty introduced by adopting both high and low construal levels is that it compels parties to think in different ways about the same information. This is also consistent with research on paradoxical cognition and creativity (Miron-Spektor et al., 2011) that suggests that considering both conflicting approaches fosters generating creative ideas. Adopting both construal levels might be a kind of desirable difficulty (Bjork & Bjork, 2011) that promotes understanding. Thus, the capability to consider both broad aims and specific details during a negotiation enables negotiators to improve their outcomes.

## 4. Discussion

### 4.1. Theoretical Implications

Negotiation scholars emphasize the importance of creativity for successful negotiation (e.g., Bazerman et al., 1985; Lax & Sebenius, 2006; Pruitt, 1983; Thompson, 1998; Walton & McKersie, 1965; Yao et al., 2020). The current studies add to a growing body of work examining negotiations in which parties can redefine the issues under discussion and so form creative agreements (Jang et al., 2021; Maddux et al., 2008; Sinaceur et al., 2013; Yao et al., 2020). This study contributes to our understanding by demonstrating that not all creative agreements are alike—unpacking issues and introducing new ones represent distinct forms of creativity. Importantly, these two types of creative outcomes arise from different cognitive processes, which are influenced by the negotiator’s thinking style (broad, specific, or both). While previous studies have typically grouped all value-creating agreements together, the results of this study suggest that the nature of the agreement is contingent upon how the negotiator is thinking.

The first experiment showed that low-level construal—a detailed, concrete thinking style—increases the likelihood of unbundling existing issues. This highlights how crucial granular attention to issue components is for identifying hidden opportunities for value creation. Prior research has often associated low-level construal with reduced creative ideation or less favorable evaluation of novel ideas (Henderson & Trope, 2009; Mueller et al., 2014; Zhbanova & Rule, 2014; Zhang et al., 2025). Detail-oriented individuals may sometimes overemphasize feasibility and convention, potentially overlooking novel ideas. While this appears to contradict the present study’s finding that detailed thinking supports unbundling—a form of negotiation creativity—it underscores that not all creativity requires abstract thought. Unbundling demands fine-grained attention to issue components, which low-level construal promotes. Thus, while abstract thinking aids in ideation and broad evaluation, concrete thinking enables structural creativity: the generation of value through the reorganization of issue elements. This finding offers a nuanced update to creativity research, highlighting the need to consider a task’s specific creative demands when applying CLT.

Experiment 2 demonstrated that negotiators capable of shifting between broad and detailed thinking—exhibiting cognitive flexibility—were more likely to reach agreements that creatively combined unbundling existing issues with introducing new ones. These findings align with Kolb’s experiential learning theory (1984), which highlights the value of fluidly moving between concrete experience and abstract conceptualization. In negotiation contexts, the ability to alternate between detailed, issue-specific analysis and a broader, abstract perspective appears to significantly enhance the generation of innovative solutions. This cognitive flexibility points to a deeper learning process that drives effective and creative problem-solving. These findings find strong resonance with the literature on desirable difficulties (Bjork & Bjork, 2011). Although encouraging negotiators to use both abstract and concrete thinking increased their cognitive load, this approach ultimately led to more comprehensive and higher-quality agreements. This aligns with the idea that cognitive strain, when well-directed, can lead to deeper processing and better problem-solving outcomes (De Dreu et al., 2008).

These findings suggest a refinement to CLT in the context of applied decision-making. While prior research has largely emphasized the advantages of high-level construals for creativity and strategic thinking (Trope & Liberman, 2010; Henderson & Trope, 2009), the current results show that this is not always the case—particularly when the creative task requires attention to detail, such as unbundling existing issues. This leads to a bigger question for CLT: When does broad thinking help and when does it hurt? The findings suggest that it is important for the way of thinking to fit the task. For complex tasks like negotiation, being able to switch between broad and detailed thinking might be more important than just thinking abstractly. Future studies could explore whether this idea applies to other creative tasks, such as strategic planning, product design, or cross-disciplinary collaboration.

These findings also provide an opportunity to reconsider the traditional negotiation principle of “focusing on interests, not positions”. While interests are usually seen as broad, abstract goals, the results of this study suggest a more nuanced perspective. Relying solely on abstract interests may not be sufficient—or even effective—when negotiating creative agreements that require adjusting the specific elements of a deal. Instead, creating value in a negotiation may require the ability to shift smoothly between different levels of abstraction: starting with broad, overarching interests and then translating them into concrete, detailed plans for the issues at hand.

This perspective offers a nuanced refinement to both negotiation theory and creativity research, which often emphasize abstract thinking as the primary driver of innovation. The findings suggest that creative solutions may stem from a dynamic interplay between abstract and concrete thinking, rather than a reliance on one cognitive style alone.

### 4.2. Implications for Management and Practice

The findings of this study have important implications for negotiation training and organizational effectiveness. First, both managers and employees could benefit from programs that cultivate integrated cognitive processing skills—enabling them to shift fluidly between abstract, strategic thinking and concrete, tactical analysis. Rather than treating these modes as distinct or mutually exclusive, individuals can be trained to flexibly apply each as needed, improving their ability to navigate complex negotiations.

Second, current negotiation practice often emphasizes goal setting and BATNA development (Bhardwaj & Sharma, 2024; Fisher et al., 2011; Lewicki et al., 2011; Mann, 2025). In addition to this, the findings of this study suggest that preparation should also include intentional mental framing exercises to activate different construal levels. For instance, teams might be asked to alternate between envisioning overarching goals and analyzing fine-grained issue components prior to negotiations.

Third, when assembling negotiation teams, managers may want to consider not only technical expertise but also cognitive style diversity. Teams with members capable of flexible thinking, or those deliberately composed of individuals with complementary construal tendencies, may be more adept at crafting creative agreements.

Lastly, outside of negotiation, these findings apply to cross-functional collaboration, problem-solving teams, and strategic planning. For example, a product manager can both articulate a high-level vision and address concrete product features. Construal flexibility may therefore be a key competence in managerial effectiveness across domains.

## 5. Conclusions, Limitations, and Future Directions

To conclude, two experiments were designed and carried out to explore how thinking abstractly versus concretely affects the development of creative solutions in negotiation. The first experiment showed that focusing on concrete details (a low construal level) helps in creating value by breaking down existing issues in new ways. The second experiment indicated that negotiators who could shift their thinking between abstract goals and concrete details were more successful in reaching agreements that maximized value. They did this both by changing existing issues and by introducing new ones, which emphasizes how important it is to be flexible in one’s thinking.

While this study gives new ideas about the thinking behind creative negotiation, there are some limits to note. First, the negotiation task in this study involved a one-time interaction between two parties. Many business deals happen in long-term relationships or involve teams not just single people. Future studies could see if being able to switch between broad and detailed thinking also helps teams or discussions that happen over time, where relationships, trust, and reputation might also influence creative results.

Second, the study mostly looked at the thinking processes that lead to creative deals. But feelings and what motivates people (like being open to new things, how much risk they take, and understanding others’ feelings) might also change how broad or detailed thinking affects creative results. Future studies could add these things to get a better overall picture.

Third, while the study observed a difference between unbundling and adding issues, it did not look at other ways to be creative in negotiation—like changing how a negotiator sees limits or using outside connections. Examining how different thinking styles help with these other ways of solving problems creatively is an important thing for future studies to do.

Lastly, while this study contributes to construal level theory by showing that high-level construals alone may be insufficient for certain forms of creativity, it does not fully unpack the conditions under which high-level abstraction becomes maladaptive. Prior research has emphasized the general benefits of abstraction for ideation (e.g., Mueller et al., 2014; Trope & Liberman, 2010; Zhang et al., 2025; Zhbanova & Rule, 2014), and this study presents a counterexample in the negotiation domain. However, more research is needed to determine whether this pattern holds in other domains or whether it is specific to negotiation tasks that require structural reconfiguration. Thus, the findings raise important questions about the boundaries of CLT’s applicability that the current design cannot entirely resolve.

For future studies, it will be interesting to see if the results of this study are the same in business deals, when groups of people negotiate, or when people from different cultures negotiate. Also, future research can look at how the ability to switch between general and detailed thinking works better over time. It will also be helpful to understand how this thinking flexibility works with feelings, motivation, or past experiences to help find creative solutions in negotiations.

Besides negotiation, these ideas can help in many areas at work. For example, good leaders and strategists need to regularly look at the big goals and also focus on the small details of how things are done. Managers also need to talk to different kinds of experts, and being able to switch between broad and detailed thinking might help them work together better and come up with new ideas. By understanding how broad and detailed thinking affects creativity, companies can create training that improves problem-solving, how to handle disagreements, and how teams work. Encouraging the ability to switch between broad and detailed thinking could be a helpful way to get better results in negotiation and to make organizations work better overall.

## Data Availability

The data presented in this study are available on request from the corresponding author because participants did not consent to public data sharing at the time of the study.
